# Radiographic Analysis of MRONJ in Osteoporotic and Oncologic Patients on Bisphosphonates or Denosumab

**DOI:** 10.1111/odi.70030

**Published:** 2025-07-14

**Authors:** Omar Ghanaiem, Gavriel Chaushu, Daya Masri

**Affiliations:** ^1^ Department of Oral and Maxillofacial Surgery, the Maurice and Gabriela Goldschleger School of Dental Medicine Tel Aviv University Tel Aviv Israel; ^2^ Department of Oral and Maxillofacial Surgery Rabin Medical Center Petah Tikva Israel

**Keywords:** bisphosphonates, denosumab, MRONJ, osteonecrosis, radiographic features

## Abstract

**Introduction:**

Medication‐Related Osteonecrosis of the Jaw (MRONJ) is a severe adverse effect of antiresorptive and antiangiogenic treatment, primarily in osteoporotic and oncologic patients. The disease is characterized by persistent jawbone necrosis that results in significant impairment of the quality of life of involved patients. The aim of this study is to compare the radiographic characteristics of MRONJ between osteoporotic and oncologic patients and assess the differences based on treatment type, particularly bisphosphonates and denosumab (RANK‐L inhibitors).

**Methods:**

A retrospective analysis of 152 MRONJ patients (41 osteoporotic, 111 oncologic) from Rabin‐Bilinson Medical Center (2014–2024) examined radiographic features—lytic changes, sclerosis, periosteal reactions, sequestration, and periodontal ligament destruction—using panoramic radiography, CBCT, and MDCT.

**Results:**

Sclerosis was more frequent in osteoporotic patients compared to oncologic patients (*p* < 0.05). No statistically significant differences were found in other radiographic features. Bisphosphonate‐induced MRONJ was more frequently accompanied by sclerosis compared to denosumab‐related cases in osteoporotic patients (*p* = 0.0318), while no significant differences were found in the oncologic group.

**Conclusions:**

Most of the radiographic features of MRONJ were similar in osteoporotic and oncologic patients. However, sclerosis was more common in osteoporotic patients exposed to Bisphosphonates, which may be due to their prolonged exposure to antiresorptive drugs.

## 1 Introduction

Medication‐Related Osteonecrosis of the Jaw (MRONJ) is a serious adverse drug effect that leads to the progressive jaw bone and soft tissue destruction. In 2014, the American Association of Oral and Maxillofacial Surgeons (AAOMS) recommended changing the acronym bisphosphonate‐related osteonecrosis of the jaw (BRONJ) to MRONJ. This revision was undertaken to consider the rising incidence of osteonecrosis of the jaw, which is linked not only to bisphosphonates but also to other antiresorptive drugs in osteoporotic and oncologic patients, as well as to antiangiogenic drugs in oncologic patients (Ruggiero et al. 2022).

MRONJ is defined by three key criteria: treatment history with antiresorptive therapy, in association with or without immune modulators or antiangiogenic agents; exposed bone or bone detectable through a fistula in the maxillofacial region persisting for over 8 weeks; and no prior radiation therapy or metastatic disease to the jaws (Ruggiero et al. 2022; Anastasilakis et al. 2022; Marx 2003). This complication can significantly impact a patient's quality of life because it commonly includes chronic pain, dysphagia, sinusitis, soft tissue abscesses, and external fistulas. MRONJ is especially severe since it mainly affects patients who are already in a vulnerable position (Filleul et al. 2010).

A staging system for MRONJ was first proposed in the 2009 AAOMS position paper and has since been altered to better reflect the clinical presentation in all its aspects (Ruggiero et al. 2022) Building on the refined staging system, AAOMS has expressed concern that placing too much emphasis on the radiographic features might lead to an over‐estimation of Stage 0 MRONJ, which presents without bone exposure and with nonspecific clinical symptoms (Ruggiero et al. 2022). The main symptoms and clinical features of MRONJ based on the AAOMS staging system are summarized in Table 1.

**TABLE 1:** symptoms and clinical features of MRONJ based on the AAOMS staging system.

MRONG stage	Symptoms and clinical features
Stage 0	No visible necrotic bone with nonspecific symptoms such as unexplained tooth pain, dull jaw pain, sinus discomfort, or altered sensation. Clinically, they may present with unexplained tooth loosening or intraoral/extraoral swelling
Stage 1	Exposed necrotic bone or probing to the bone fistula in asymptomatic patients without signs of infection or inflammation
Stage 2	Exposed necrotic bone or probing to the bone fistula in symptomatic patients with signs of infection or inflammation
Stage 3	Exposed necrotic bone or probing to the bone fistula in symptomatic patients with signs of infection or inflammation. And one or more of the following: necrosis beyond the alveolar bone, pathologic fractures, extraoral fistulas, oral‐antral or oral‐nasal communication, and extensive osteolysis reaching the mandible's inferior border or sinus floor

MRONJ occurs much less often in osteoporotic patients than in oncologic patients. According to the latest consensus (Ruggiero et al. 2022), the incidence of Bisphosphonate Related Osteonecrosis of the Jaw (BRONJ) in osteoporosis patients ranges between 0.01% to 0.06%, while osteoporotic patients receiving denosumab were 37% less likely to develop denosumab related osteonecrosis of the jaw (DRONJ) than untreated patients (odds ratio: 0.63, *p* < 0.001) (Zhang et al. 2016). In contrast, oncologic patients face a much higher risk; the incidence of BRONJ following zoledronic acid (ZOL) treatment varies, ranging from 1% to 8% in patients treated for bone metastases and from 0% to 1.8% when used as an adjuvant therapy (6). A key reason for this difference is the significantly higher dose of bisphosphonates and denosumab used for metastatic bone disease compared to osteoporosis (Anastasilakis et al. 2022).

**FIGURE 1:**
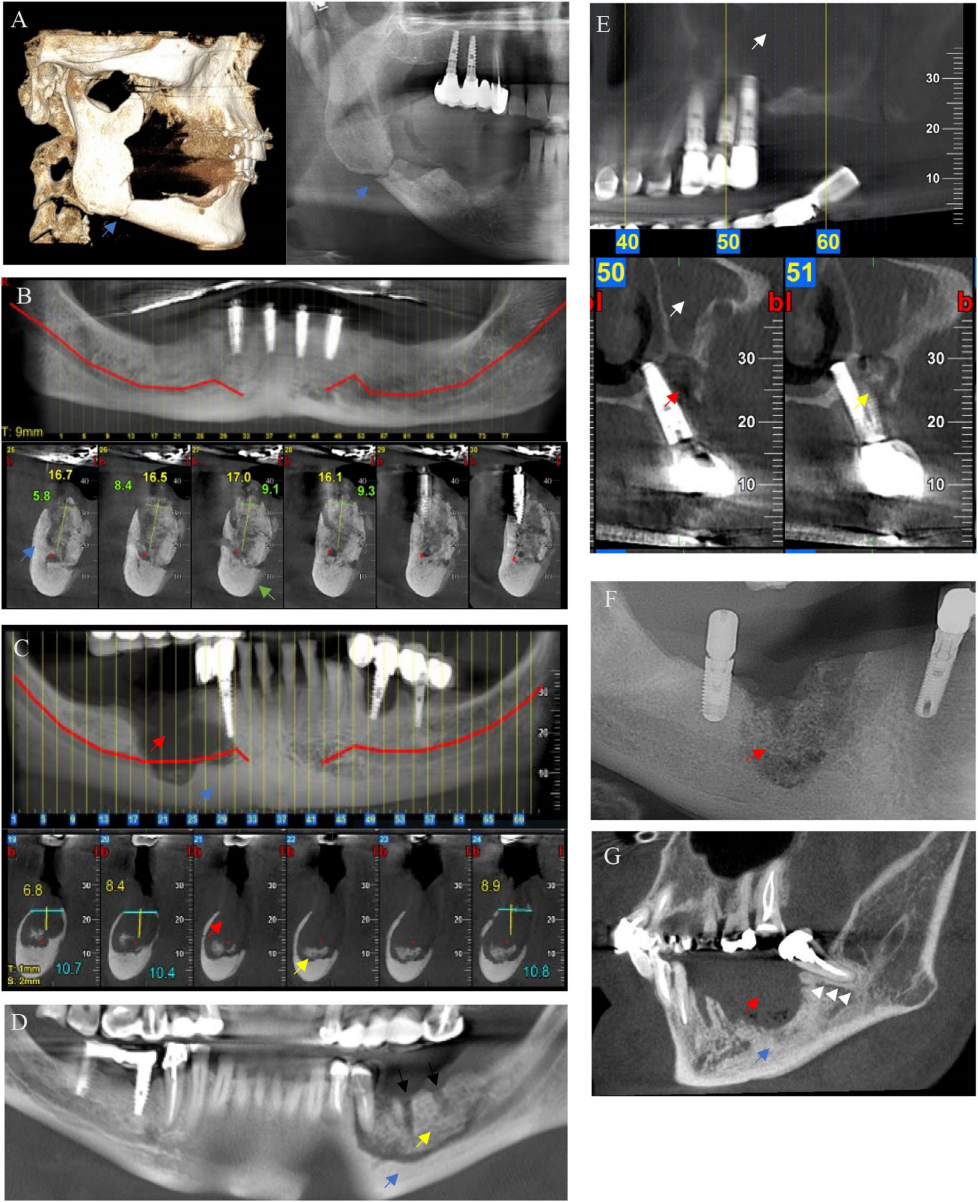
Representative radiographic features of MRONJ. (A) Right mandibular pathological fracture (blue arrow); left: Three‐dimensional MDCT reconstruction, right: Panoramic radiograph. (B) Right mandibular sclerosis (blue arrow), lytic lesion (red arrow), and subperiosteal reaction (green arrow); upper: Panoramic CBCT reconstruction, lower: Axial CBCT sections. (C) Extensive right mandibular lysis (red arrow), sclerosis (blue arrow), and sequestration (yellow arrow); upper: Panoramic CBCT reconstruction, lower: Axial CBCT sections. (D) Left mandibular sequestration (yellow arrow), sclerosis (blue arrow), and unhealed extraction sites (black arrows); panoramic CBCT reconstruction. (E) Left maxillary sequestration (yellow arrow), sclerosis (red arrow), and maxillary sinus opacification (white arrow); upper: Panoramic CBCT reconstruction, lower: Axial CBCT sections. (F) Extensive right mandibular lysis (red arrow); panoramic CBCT reconstruction. (G) Extensive left mandibular lysis (red arrow), sclerosis (blue arrow) and thickening of the lamina dura (white arrow heads); MDCT.

Some of the most common radiographic features of MRONJ include lytic change, sclerosis, periosteal bone formation, sequestration, thickening of the lamina dura, and destruction of the periodontal ligament (Figure 1) (Shin et al. 2024).

In MRONJ, sclerosis is seen as increased bone density due to suppressed bone turnover from antiresorptive medications and chronic inflammation, and lysis involves bone destruction resulting from progressive necrosis and secondary infection (Obermeier et al. 2024) (Zhang et al. 2016). In addition, as a response to this abnormal stimulus, a periosteal reaction may also be seen as a nonspecific radiologic finding that demonstrates new bone formation from the abnormal stimuli (Soutome et al. 2021) (Figure 1).

If bone destruction occurs, a bony sequestrum—essentially a fragment of dead bone—can form as it separates from the surrounding normal bone during necrosis. This is typically found in pathology like bone infection, such as osteomyelitis. A radiographic bony sequestrum can be noted as a calcified tissue surrounded by a darker, lucent zone (Jennin et al. 2011).

Lamina dura thickening seen in MRONJ patients is usually the sequelae of altered bone remodeling due to drugs like bisphosphonates and denosumab. These drugs slow down the activity of osteoclasts, the cells responsible for breaking down old bone, which causes the bone to become denser and more sclerotic. This change can also be observed on X‐rays, as the lamina dura becomes denser and more visible, representing a non‐specific characteristic of bone alterations in stage 0 MRONJ. These radiographic changes are an early clue to how the jawbone is affected in patients on these therapies (Anastasilakis et al. 2022; Kim et al. 2020).

Walton et al. (2019) conducted a comparative study to assess the radiographic characteristics of MRONJ in 29 osteoporotic versus 41 oncologic patients, using CBCT imaging in order to assess the distinctive radiographic characteristics of MRONJ. They reported no statistically significant differences in sclerosis, lytic changes, sequestration, or periosteal reaction between osteoporotic and oncologic patients. (Walton et al. 2019).

In a more recent study (Shin et al. 2024), Shin et al. aimed to compare the clinical and radiological features of MRONJ in osteoporotic versus oncologic patients and clarify the differentiating aspects. From cone‐beam computed tomography (CBCT) and/or multidetector row computed tomography (MDCT) and/or panoramic radiography images. In their results, the radiographic features of (Trabecular defect and sclerosis cortical defects and sequestration) were comparable in both patient groups (Shin et al. 2024).

Given the incidence of MRONJ in osteoporotic and oncologic patients and its significant burden on both patients and the healthcare system, our study aimed to explore the radiographic differences between these two groups. By identifying these differences, we aim to enable early diagnosis, permit early intervention, and prevent the need for complex procedures, and ultimately decrease the burden on both patients and healthcare providers (Yarom et al. 2019; Thomas and Ouanounou 2023).

## 2 Material and Methods

### 2.1 Sample Population and Data Collection

With the approval of the Research Helsinki Committee at Rabin‐Bilinson Medical Center (RMC‐0160‐23), we conducted a retrospective analysis of MRONJ patients monitored at the Department of Oral and Maxillofacial surgery at Rabin‐Belinson medical Center between 2014 and 2024. The study included individuals who received antiresorptive or antiangiogenic therapy for osteoporosis or oncological conditions and subsequently developed jaw necrosis as a result of the treatment.

The inclusion criteria included:

1. Oncologic or osteoporotic patients treated with antiresorptive therapy.
2. Patients diagnosed with MRONJ.
3. Available radiographic and clinical follow‐up.

The exclusion criteria included:

1. Patients with a history of head and neck radiation.
2. Oncologic or osteoporotic patients treated with antiangiogenic therapy.
3. Insufficient follow‐up.
4. Inadequate radiographs.

Demographic data, medical background, and dental imaging were collected, including cone‐beam computed tomography (CBCT), multi‐detector computed tomography (MDCT), and simple planar imaging such as panoramic X‐rays or periapical X‐rays. Additionally, various characteristics related to MRONJ were documented, including the severity of the disease (AAOMS staging), the type of drug treatment (bisphosphonates vs. denosumab), duration of exposure (years), treatments provided (ranging from conservative approaches, such as mouthwash and antibiotics, to curettage and sequestration, partial resection of the affected jaw [marginal resection], and complete resection [segmental resection]), and the final outcome (disease resolution).

For patients with appropriate imaging, a systematic analysis was performed by two senior maxillofacial surgeons (D.M. and G.C.) in the department of Oral and Maxillofacial Surgery to identify predetermined radiographic features, including lytic changes, sclerosis, periosteal bone formation, sequestration, thickening of the lamina dura, and destruction of the periodontal ligament (PDL). The analysis also included the distribution of the disease between the jaws (maxilla vs. mandible) and among different areas of the jaw (anterior region between the canines, premolar region, and molar region).

### 2.2 Statistical Analysis

Patient characteristics were summarized using frequencies (percentages) or means (standard deviations), unless otherwise specified. To compare characteristics between the osteoporotic versus oncologic groups and Bisphosphonates versus denosumab groups, the t‐test was used for age, while Chi‐square or Fisher's exact test was applied to categorical variables as appropriate. Statistical analyses were conducted using IBM SPSS. A *p* value of < 0.05 was considered statistically significant.

## 3 Results

The study group consisted of 221 patients who developed jaw necrosis after the intake of antiresorptive or antiangiogenic medication. Among them, 67 patients had osteoporosis as the underlying disease, and 145 patients had an oncological condition at the time of MRONJ diagnosis. In the osteoporosis group, 41 patients with complete follow‐up and imaging were present. In the oncology group, 111 patients met the imaging criteria. Consequently, the study population comprised 152 patients.

**FIGURE 2:**
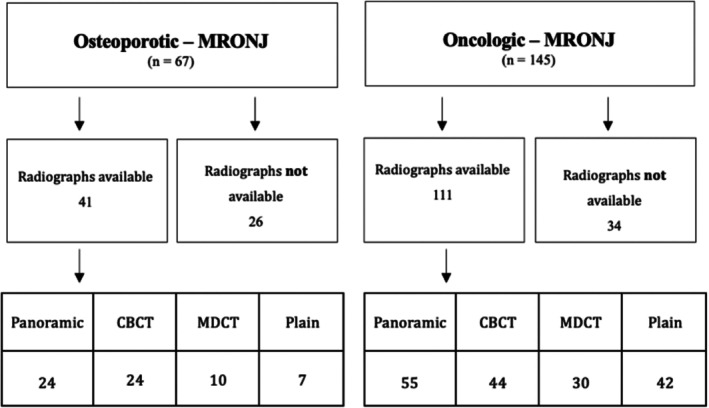
Study population and radiographs available.

Most of the participants in the study had at least one type of imaging. In the osteoporotic group, there were 24 panoramic radiographs, 24 dental CT scans, 10 multi‐detector CT scans, and 7 planar radiographs. While in the oncology group, there were 55 panoramic radiographs, 44 CBCT scans, 30 multi‐detector CT scans, and 42 planar radiographs. (Figure 2).

In the osteoporotic group, a total of 41 patients were included, with an average age of 74.22 ± 9.52 years. The group consisted of 5 men and 36 women. Among these patients, 33 (80%) were treated with bisphosphonates, while 8 (20%) were treated with denosumab, with an average treatment duration of 7.4 years (range: 2–20 years). The majority of MRONJ cases were diagnosed at advanced stages, with 22 patients (54%) classified as Stage 2, 15 patients (36%) as Stage 3, and 4 patients as Stage 1. Most cases were localized in the mandible, predominantly in the molar region (24 cases, 60%) and the premolar region (14 cases, 34%). A total of 23 cases were treated with conservative removal of the bone sequestrum, while 15 cases underwent partial resection of the jaw. Overall, a significant proportion of patients (83%) achieved full recovery from the disease (Table 2).

**TABLE 2:** Demographic and clinical findings of medication‐related osteonecrosis of the jaw (MRONJ) in the osteoporotic and oncologic groups.

	Osteoporotic group	Oncologic group	*p*
Number	41	111	
Gender (Male: Female)	5:36	55:56	**0.0000**
Age (mean ± SD)	74.22 ± 9.52	73.56 ± 12.40	0.7286
Anti‐resorptive treatment			
Medication duration (years ± SD) (range)	7.4 ± 5.21 (range 2–20)	2.7 ± 2.07 (range 0.5–14)	**0.0000**
Bisphosphonates	33 (80%)	75 (68%)	**0.0000**
Denosumab	8 (20%)	33 (30%)	**0.0000**
Both	0	3 (2%)	0.564
Location^a^			
Maxilla: Mandible	7:34	18:93	1.0
Anterior region	14 (34%)	20 (18%)	**0.0048**
Premolar region	14 (34%)	58 (52%)	0.067
Molar region	24 (60%)	86 (77%)	**0.025**
Paresthesia complain	10 (24%)	24 (21%)	0.827
MRONJ staging			**0.0012**
Stage 0	1 (2%)	2 (2%)	1.0
Stage 1	3 (8%)	31 (30%)	**0.0076**
Stage 2	22 (54%)	65 (58%)	0.712
Stage 3	15 (36%)	13 (10%)	**0.0008**
Definitive Treatment^b^			
Antibiotics and CHx rinse	2 (5%)	47 (42%)	**0.0000**
Sequestrectomy	23 (56%)	51 (45%)	0.279
Marginal resection	15 (36.5%)	12 (11%)	**0.0000**
Segmental resection	1 (2.5%)	1 (1%)	0.468
Disease resolution	34 (83%)	78 (70%)	0.147

*Note:* the bold values are the values who are significant (*P <* 0.05).

^a^ Some of the lesions were in multiple regions in the jaws.

^b^ Some of the patients were treated with multiple modalities.

Among the oncologic group, a total of 111 patients were included, with an average age of 73.56 ± 12.40 years. The group comprised 55 men and 56 women. Of these, 75 patients (68%) were treated with Bisphosphonates, and 33 patients (30%) received denosumab for an average duration of 2.7 years (range: 0.5–14 years). Most cases were in intermediate stages, with 65 patients (58%) diagnosed as stage 2, 31 patients (30%) as stage 1, and 13 patients (10%) as stage 3. Most of the cases were localized in the mandible, primarily in the molar region (86 cases, 77%) and the premolar region (58 cases, 52%). A total of 47 cases were managed with conservative treatment, including medication, while 51 cases underwent conservative sequester removal. Overall, a high success rate was observed, with 70% of patients achieving complete resolution of the disease. (Table 2).

The anti‐resorptive treatment consisted of two groups of drugs. The first group included bisphosphonates, such as (Fosamax, Actonel, Reclast, Boniva), while the second group included denosumab (anti‐RANK‐L agents), such as (Prolia). In the oncology group, 75 patients were treated with bisphosphonates, 33 patients received denosumab, and only 3 patients were treated with both types of drugs (bisphosphonates and denosumab). In the osteoporotic group, 33 patients received bisphosphonates, while only 8 patients were treated with denosumab. A statistical test assessing the distribution of drugs within each group demonstrated a statistically significant difference (*p* < 0.001), indicating that the use of bisphosphonates is more prevalent compared to the use of denosumab.

In both the osteoporotic and oncologic groups, the majority of patients were diagnosed with grade 2 MRONJ at the time of admission. Specifically, in the oncologic group, the distribution of MRONJ stages was as follows: 2 patients at stage 0, 31 patients at stage 1, 65 patients at stage 2, and 13 patients at stage 3. Similarly, in the osteoporotic group, the distribution was: 1 patient at stage 0, 3 patients at stage 1, 22 patients at stage 2, and 15 patients at stage 3.

When comparing the various radiographic findings between the oncologic group and the osteoporotic group (Table 3), it was observed that among 111 oncologic patients diagnosed with jaw necrosis, 94 exhibited lytic lesions, 47 had sequestration, 43 showed sclerotic lesions, 32 presented with involvement of the inferior alveolar nerve, and 19 had periosteal reactions. In contrast, among 41 osteoporotic patients with jaw necrosis, 40 exhibited lytic lesions in the jaws, 26 showed sclerosis, 22 had sequestration, 10 demonstrated periosteal reactions, and 18 had involvement of the mandibular canal.

**TABLE 3:** Radiographic features in oncologic versus osteoporotic group.

Radiographic lesion	Oncologic group (*N* = 111)	Osteoporotic group (*N* = 41)	Chi^2^ statistic	*p*
Lytic change	94	40	2.1942	0.138
Sclerosis	43	26	6.1286	**0.013**
Periosteal bone formation	19	10	0.8087	0.368
Sequestration	47	22	1.0644	0.302
Thickening of lamina dura	8	1	1.3183	0.250
Destruction of the (PDL)	7	2	0.1452	0.703
Pathological fracture	4	3	0.8356	0.360
Involvement of the inferior alveolar nerve	32	18	2.511	0.113
Maxillary sinus involvement	7	1	0.9972	0.317

*Note:* the bold values are the values who are significant (*P <* 0.05).

In a statistical analysis using the Chi‐square test to compare the radiographic findings between the two groups, a statistically significant difference was observed in sclerosis. A higher percentage of osteoporotic patients exhibited bone sclerosis compared to oncologic patients (*p* < 0.05).

In order to assess the influence of the type of drug treatment on the radiographic appearance of MRONJ, both patient groups were further divided into one treated with Bisphosphonates and the other treated with denosumab. Among oncologic patients, no statistically significant difference was observed in the radiographic appearances between the two drug types (Table 4). On the other hand, in the osteoporotic group, a statistically significant difference was found in the radiographic appearance (sclerosis), which was considerably more frequent in the Bisphosphonate group compared to the denosumab group (Table 5). To compensate for the limited sample size, a consolidation of the two samples was performed, and on statistical analysis, no significant difference was noted between the Bisphosphonate group and the denosumab group (Table 6).

**TABLE 4:** Comparison between bisphosphonates versus denosumab groups in osteoporotic patients.

Radiographic lesion	Bisphosphonates (*N* = 33)	Denosumab (*N* = 8)	Chi^2^ statistic	*p*
Lytic change	30	8	0.0335	0.8547
Sclerosis	22	2	4.6058	**0.0318**
Periosteal bone formation	8	2	0.002	0.9642
Sequestration	19	3	1.0437	0.3069
Thickening of lamina dura	0	1	1.3048	0.2533
Destruction of the (PDL)	2	0	0.3937	0.5303
Pathological fracture	3	0	0.085	0.7706
Involvement of the inferior alveolar nerve	14	3	0.0643	0.7997
Maxillary sinus involvement	1	0	1.244	0.2646

*Note:* the bold values are the values who are significant (*P <* 0.05).

**TABLE 5:** Comparison between bisphosphonates versus denosumab groups in oncologic patients.^a^

Radiographic lesion	Bisphosphonates (*N* = 75)	Denosumab *N* = 33	Chi2 statistic	*p*
Lytic change	59	29	0.8028	0.3705
Sclerosis	29	12	0.1083	0.742
Periosteal bone formation	11	6	0.1636	0.6858
Sequestration	25	12	0.0448	0.8323
Thickening of lamina dura	4	2	0.0144	0.9045
Destruction of the (PDL)	3	2	0.1925	0.6608
Pathological fracture	2	2	0.6902	0.4060
Involvement of the inferior alveolar nerve	23	7	1.187	0.2759
Maxillary sinus involvement	3	4	2.3651	0.1240

^a^ Three patients consumed both bisphosphonates and denosumab were excluded.

**TABLE 6:** Comparison between bisphosphonates versus denosumab groups in all patients.^a^

Radiographic lesion	Bisphosphonates (*N* = 106)	Denosumab (*N* = 41)	Chi^2^ statistic	*p*
Lytic change	89	37	0.9527	0.329
Sclerosis	51	14	2.3382	0.1262
Periosteal bone formation	19	8	0.0497	0.8235
Sequestration	44	15	0.2983	0.5849
Thickening of lamina dura	4	3	0.8185	0.3656
Destruction of (PDL)	5	2	0.0017	0.9671
Pathological fracture	5	2	0.0017	0.9671
Involvement of the inferior alveolar nerve	37	10	0.0292	0.8644
Maxillary sinus involvement	4	4	2.0562	0.1515

^a^ Three patients consumed both bisphosphonates and denosumab were excluded.

## 4 Discussion

### 4.1 Demographic and Clinical Findings

This article provides a comparative analysis of osteoporotic and oncologic patients with MRONJ, emphasizing key radiographic differences between these two groups, as well as between patients treated with bisphosphonates and those treated with denosumab. A significant gender difference was observed (*p* < 0.0001), with a higher proportion of female patients in both groups. Several studies support the observed gender differences in MRONJ prevalence between osteoporotic and oncologic patients. Dioguardi et al. (2023) found osteoporotic MRONJ to largely occur among elderly females, reflecting the high prevalence of osteoporosis in postmenopausal women. Oncologic MRONJ cases exhibit a more balanced gender distribution due to the widespread use of antiresorptive therapy in both male and female oncologic patients. However, age distribution was not significantly varied between groups (*p* = 0.7286).

One of the most noticeable differences was the duration of anti‐resorptive treatment, with much longer exposure in the osteoporotic group (7.4 ± 5.21 years vs. 2.7 ± 2.07 years, *p* < 0.0001). This aligns with Joan et al. (Lo et al. 2010), who identified that the incidence of MRONJ was higher among patients exposed more than 4 years than among patients with exposure less than 4 years (0.21% vs. 0.04%, respectively, *p* = 0.03). These results provide a compelling explanation for our results, particularly the significantly higher prevalence of sclerosis in osteoporotic patients compared to oncologic patients. A prolonged exposure to antiresorptive drugs leads to a greater reduction in bone remodeling, ultimately contributing to a more pronounced sclerotic appearance.

### 4.2 MRONJ Location and Staging

The clinical, radiographic diagnosis and staging of MRONJ was determined following the guidelines established by the American Association of Oral and Maxillofacial Surgeons (AAOMS) (Ruggiero et al. 2022).

Our study found that lesions were more common in the mandible than the maxilla, but the difference was not statistically significant (*p* = 1.0). This finding aligns with Saad et al. (2012) and Hallmer et al. (2018), who explained that more vascularity within the maxilla may reduce the risk of MRONJ compared to the mandible. However, lesion distribution by region showed significant differences: anterior region lesions were more common in osteoporotic patients (*p* = 0.0048), while molar region lesions were significantly more common in oncologic patients (*p* = 0.025).

MRONJ staging was profoundly different (*p* = 0.0012). Stage 1 was significantly more common in oncologic patients (*p* = 0.0076), perhaps due to earlier detection by routine oncologic follow‐ups. Stage 3 was significantly more common in osteoporotic patients (*p* = 0.0008), supporting findings by Anastasilakis et al. (2022), who reported that osteoporotic patients are often diagnosed at later stages due to delayed symptom recognition and limited awareness among general practitioners regarding this type of lesion.

### 4.3 Treatment Modalities and Outcomes

Treatment protocols differed significantly; conservative treatment with antiseptic mouth rinses (chlorohexidine) and systemic antibiotic treatment were more commonly used in oncologic patients (*p* < 0.0001), reflecting a preference for conservative management in early‐stage MRONJ. On the other hand, marginal resection was significantly more frequent in osteoporotic patients (*p* < 0.0001). Anastasilakis et al. (2022) may indirectly support these findings in their article, Oncologic patients are more likely to be diagnosed with MRONJ earlier since they are followed up more frequently, especially during cancer treatments. This early diagnosis allows for a more conservative management approach. Osteoporotic patients, on the other hand, are likely to be diagnosed late since they typically receive antiresorptive therapy for longer periods but are less intensively kept under check. This delayed diagnosis can lead to advanced stages of MRONJ, necessitating surgical interventions like marginal resection (Anastasilakis et al. 2022).

Interestingly, disease resolution rates did not differ significantly (83% in osteoporotic vs. 70% in oncologic, *p* = 0.147). Data directly comparing MRONJ resolution rates between osteoporotic and oncologic patients are limited. Wutzl et al. (2012) compared the benefits of surgery for MRONJ in osteoporotic versus oncologic patients and concluded that osteoporotic patients achieved better improvement post‐surgery than those with cancer. On the other hand, Vahtsevanos et al. (Vahtsevanos et al. 2009) suggested that the better surgical outcomes in osteoporotic patients might be due to differences in preoperative staging, making them more responsive to treatment than cancer patients (Ruggiero et al. 2022).

Although not significantly statistical, disease resolution happened more among the osteoporotic patients compared to oncologic patients (83% versus 70% respictevly). Additionally, osteoporotic patients were also diagnosed at an advanced stage compared to oncologic patients. Therefore, from what we have concluded, resolution is more prevalent among the osteoporotic patients. But this assumption conflicts with that of Vahtsevanos et al. (Vahtsevanos et al. 2009), who associated better prognosis with earlier staging in osteoporotic patients, but agrees with Wutzl et al. (2012), who came to the conclusion that osteoporotic patients improved better after surgery in comparison with oncologic patients.

### 4.4 Radiographic Features in MRONJ

Radiographic findings play a crucial role in diagnosing and MRONJ patient follow‐up, mainly for differentiation of patient groups and disease progression (He et al. 2020). This investigation analyzed radiographic differences between osteoporotic and oncologic patients and Bisphosphonates versus denosumab receiving patients.

#### 4.4.1 Comparison Between Oncologic and Osteoporotic Patients

Of all the radiographic parameters evaluated, the lone parameter that recorded statistically significant disparity in osteoporotic and oncologic patients was sclerosis (*p* = 0.013), being more common in the osteoporotic group. In contrast, Jong Mon et al. (Shin et al. 2024) noted that there are no notable differences in radiographic signs between the osteoporotic and oncologic groups. Osteoporotic patients typically receive lower doses over an extended period of time to inhibit bone resorption and reduce fracture risk. In contrast, oncologic patients typically receive more aggressive doses for the treatment of bone metastases and malignancy‐related hypercalcemia. Prolonged bisphosphonate administration can lead to oversuppression of bone remodeling, which potentially is a component of increased sclerosis observed in radiographic assessments (Kennel and Drake 2009).

Other radiographic lesions, including lytic changes, sequestration, periosteal bone formation, pathological fracture, and inferior alveolar nerve involvement, showed no significant differences between groups.

#### 4.4.2 Bisphosphonates Versus Denosumab in Radiographic Presentation

To the best of our knowledge, our study is the first to contrast the radiographic presentation of MRONJ in bisphosphonate‐treated and denosumab‐treated patients within both oncologic and osteoporotic populations. In oncologic patients, no significant differences between bisphosphonate and Denosumab users existed for any radiographic feature, whereas in osteoporotic patients, sclerosis was much more frequent among bisphosphonate users compared to Denosumab users (*p* = 0.0318). This is possibly due to the fact that bisphosphonates incorporate into the bone matrix, leading to increased mineralization and sclerosis over time, whereas denosumab acts by directly inhibiting osteoclast activity, leading to more rapid bone remodeling and resorption (Ruggiero et al. 2022). Further detailed comparison between bisphosphonates and denosumab across all study groups revealed no radiographic features with significant difference.

Our findings share several similarities with the study by Walton et al. (2019), who also compared radiographic features of MRONJ in osteoporotic and oncologic patients. Both studies observed that most radiographic features—including lytic changes, sequestration, and periosteal reaction—did not significantly differ between patient groups. However, Walton et al. (2019) did find, however, bisphosphonate‐treated patients showed greater overall radiographic disease burden than denosumab‐treated patients, likely due to the longer skeletal half‐life and cumulative effects of bisphosphonates. Consistently, our results showed a greater prevalence of sclerosis in bisphosphonate‐treated osteoporotic patients compared to those treated with denosumab. Our study included a larger cohort and combined CBCT, MDCT, and panoramic imaging, allowing for a broader analysis of maxillary and mandibular lesions.

### 4.5 Limitations and Future Directions

While our study provides valuable insights, a number of limitations should be noted:

- Retrospective Design: Our analysis relied on retrospective radiographic assessments, which may introduce bias due to variability in imaging techniques.
- Sample Size: The relatively small number of denosumab users in the osteoporotic group may have affected statistical power.
- Lack of Longitudinal Data: Future studies should track radiographic changes over time to better understand MRONJ progression in different patient populations.
- Low number of MRONJ cases involving the maxilla compared to the mandible. Even within this large cohort, this imbalance limited our ability to draw definitive conclusions about specific radiographic changes and differences in maxillary lesions. To better understand maxillary MRONJ and improve diagnostic accuracy, future multicenter studies focusing exclusively on maxillary cases are warranted.

## 5 Conclusion

Our research highlights significant radiographic differences between oncologic and osteoporotic MRONJ patients, where higher sclerosis frequency is found in osteoporotic patients. We further noted that bisphosphonate‐induced MRONJ is more likely to present with sclerosis when compared to denosumab‐induced MRONJ. These findings emphasize the importance of radiographic evaluation in MRONJ diagnosis, staging, and treatment planning, reinforcing the need for customized management strategies based on drug exposure history and radiographic presentation.

## Author Contributions

**Omar Ghanaiem:** conceptualization, investigation, funding acquisition, writing – original draft, writing – review and editing, visualization, validation, methodology, software, project administration, formal analysis, resources, supervision, data curation. **Gavriel Chaushu:** resources, supervision, data curation, software, formal analysis, project administration, writing – review and editing, visualization, validation, methodology, conceptualization, investigation, funding acquisition, writing – original draft. **Daya Masri:** writing – original draft, funding acquisition, investigation, conceptualization, methodology, validation, visualization, writing – review and editing, project administration, formal analysis, software, resources, supervision, data curation.

## Conflicts of Interest

The authors declare no conflicts of interest.

## Data Availability

The data that support the findings of this study are available from the corresponding author upon reasonable request.
